# Lunatic Asylums in Ireland

**Published:** 1851-10-01

**Authors:** 


### Art. III.—LUNATIC ASYLUMS IN IRELAND.

We have before us the fifth general report of the *District, Criminal, and Private Lunatic Asylums in Ireland*, signed by Francis White and John Nugent, Esqrs., the official inspectors of lunatics. It is an elaborate and important document, and appears to be drawn up with great care and ability. The last report was presented at the close of the session of 1849. The additional asylums ordered by the Lord-Lieutenant to be erected for the accommodation of the insane poor, subsequent to the presentation of the parliamentary return of 1849, are not, it appears, yet fit for the habitation of the patients. Towards the close of the year, it is hoped that the Cork, Kilkenny, Omagh, and Killarney Institutions, adapted for 1100 patients, inclusive of the asylums building at Sligo and Mullingar for 520, will be finally completed.

The Richmond or Metropolitan Institution has undergone an enlargement within the last twelve months for the reception of 140 patients, with the addition of a spacious kitchen, laundry, and range of out-offices, at the same time that thirty acres adjoining the former grounds have been purchased, on which a new hospital, furnished with 160 beds, and especially appropriated to the treatment of mental disease in its early stages, is about to be erected.

The district asylum at Ballinasloe, for the province of Connaught, has been materially increased by the extension of two wings for ninety additional patients, with commodious day-rooms and workshops, as also by the erection of an infirmary, kitchen, wash-house, and out-offices. The farm, increased by a late purchase of twenty, now contains fifty statute acres; whilst the airing yards, in consequence of the removal of gloomy enclosures which had before materially interfered with light and ventilation, are rendered more open and ventilated. This, with the asylum at Sligo for 250 patients when finished, is calculated to meet the existing necessities of Connaught, with regard to its destitute insane.

The Belfast institution is at present not only amply supplied with land for farming purposes, but secure from the too close approach of factories or private dwellings, by the possession and enclosure last year of certain fields containing fourteen acres, which lay between it and the town.

Considerable ameliorations have been further effected in the Clonmel district asylum, for the east and west riding of Tipperary, by the addition of an infirmary, bath-room, lavatories, &c. &c. The resident physician and superintendent, Dr. Flynn, is desirous of an extension of land, finding that the present quantity is not sufficient to afford occupation to the inmates, who belong altogether to the agricultural community. The inspectors having been long of this opinion, have recommended to the governors the utility and economy of Dr. Flynn's proposition, and arrangements are being made with a view of carrying it into immediate effect.

The Limerick asylum is now surrounded by a well-enclosed farm of 36 acres, which affords a full opportunity for remunerative out-door employment. The acquisition of the ground, not only at its western or city side and contiguous to the gaol, but of that adjoining the railroad, protects the institution, as at Belfast, from being interfered with by private buildings.

In speaking of the general management of these asylums, we are gratified to hear the inspectors observe—

"We have reason to express satisfaction at the general arrangements and domestic economy of these institutions. Constant in our visitations of them, we invariably observe the utmost kindness of manner and considerate attention on the part of the physicians and superior officers to the various inmates, while the attachment of the lunatics themselves to their immediate attendants, affords a satisfactory proof that the latter fulfil their duties with humanity, good temper, and forbearance—moral powers, for which mechanical coercion will be ever found from experience both a harsh and inefficient substitute. This social condition of our public asylums, coupled with the attention bestowed on them by the governors, who are appointed for the most part from amongst the principal proprietors in the respective districts, for fiscal and general purposes, cannot fail to place them ultimately on a rank with the first establishments of the kind in any country."

The following quotation will convey to our readers a correct idea of the present amount of insanity existing in Ireland:—

"1. In public and local asylums . . . . . . . . . . . . . . . 2913
2. In gaols, committed as dangerous, 1 Vic., cap. 27 . . . . . . . 280
3. In central asylum, Dundrum, 8 and 9 Vic., cap. 57 . . . . . . . 91
4. In poor-houses . . . . . . . . . . . . . . . . . . . . 2393
5. In private asylums, 5 and 6 Vic., cap. 123 . . . . . . . . . . 436
6. Abroad, unprovided for in public institutions, but some supported by their friends . . . . . . . . . . . . . . . . . . . . 8985"

The subjoined tables refer to the "unaccommodated insane":—

| Idiots. | | Epileptic Imbeciles. | | Lunatics. | |
|---|---|---|---|---|---|
| Male . . . | 1990 | Male . . . | 1644 | Male . . . . | 452 |
| Female . . | 1684 | Female . . | 2736 | Female . . . | 479 |
| Total . . | 3674 | Total . . | 4380 | Total . . | 931 |

"A total as above stated of 8985, consisting of 4086 males and 4899 females.

Adopting the same classification for the 2393 deranged inmates of unions and auxiliary workhouses, we find them thus circumstanced:—

| Idiots. | | Epileptic Imbeciles. | | Lunatics. | |
|---|---|---|---|---|---|
| Male . . . | 471 | Male . . . | 350 | Male . . . | 77 |
| Female. . . | 645 | Female . . . | 739 | Female . . . | 111 |
| Total . . | 1116 | Total . . | 1089 | Total . . | 188 |

Or, 898 males and 1495 females.

Three-fourths of the inmates at present in the Irish district establishments—and we believe the same observation applies to many in England—are the accumulation of chronic disease, with little or no prospect of recovery. As illustrative of this position, we may instance the asylum at Clonmel, with the duration of residence of the 128 patients it contains, 71 of whom are there over five years:—

| Over 15 years. | | Over 10 and under 15 years. | | Over 5 and under 10 years. | | Over 1 and under 5 years. | | One year and under. | |
|---|---|---|---|---|---|---|---|---|---|
| M. | F. | M. | F. | M. | F. | M. | F. | M. | F. |
| 12 | 6 | 10 | 12 | 12 | 19 | 19 | 20 | 11 | 7 |
| 18 | | 22 | | 31 | | 39 | | 18 | |

It would be an injustice akin to cruelty to discharge unhappy beings circumstanced as the great majority of the above are, without first providing an abode where means of employment and every reasonable comfort, adapted to their mental condition, would be afforded them. At the same time it is quite evident that the public are wronged by the appropriation of institutions, constructed and upheld at great expense, to purposes different from those for which they were intended—namely, as curative hospitals; and that the insane themselves not only suffer from the want of early treatment, but in numerous instances are thereby rendered perpetual burdens on the country, as exemplified at Clonmel, where, under existing circumstances, the recoveries do not exceed 20 per cent. per annum, on the aggregate number of patients, leaving thus a difference of 40 between the common average of cures, when admission is obtained in the first stages of disease.

If the sole difficulty with regard to future arrangements depended on the removal from asylums of cases when they became chronic, and on their subsequent disposal, it might be easily dealt with; but independent of incurables who crowd the Irish district institutions, and the lunatics committed to gaols, there is a mass of insanity, in all varieties of form, to be found in poor-houses, as well as amongst the "unprovided for abroad," consisting principally of idiots, imbeciles, epileptics, and individuals bordering on fatuity, for the vast proportion of whom the cost of building and maintaining regularly constructed asylums, such as at present exist, would be an utter waste of expenditure. At the same time we are fully satisfied that, at no distant period, accommodation must be prepared for the unhappy classes just referred to.

The poor-houses of Ireland contain 2393 insane persons of every degree and denomination, each house averaging about 20 inmates. In speaking of the cost of maintenance of lunatics, the inspectors observe—

"The total cost of maintenance to the country of the destitute insane, as liquidated by grand jury presentments, and repaid to the treasury for quarterly advances, 1st & 2nd Geo. IV. c. 33, has progressively declined for the last four years. In 1847 it amounted to 46,736*l.* 16*s.* 11*d.*; in 1848, 42,491*l.* 12*s.* 8*d.*; in 1849, 40,956*l.* 17*s.*; and in 1850, to 37,252*l.* 11*s.*; the average number of patients being the same—about 2730. This decrease of expense has not, however, been alike in all asylums; in some it is more marked than in others, a circumstance depending on the relative cost of provisions, and a difference of dietary. As this variation of dietary is not desirable, we purpose submitting to the different boards of governors a uniform scale, which we trust will meet their approbation; for on all occasions we have found them the willing and liberal supporters of any proposition tending to the comforts of the insane; and it affords us much gratification to state that the utmost cordiality exists, not only between them and the inspectors, but also the various officers connected with these great public institutions."

It is gratifying to find by the report before us, that, in a curative point of view, the Irish district asylums offer a favourable contrast to similar establishments in other countries; the proportion of recoveries on recent admissions, if it does not surpass, fully equals the average elsewhere—a result materially accruing from good air, exercise, and outdoor employment, while from similar causes, and judicious medical treatment, notwithstanding the prevalence of epidemics, and the accumulation of aged and chronic cases, the mortality of the last two years has been sensibly diminished.

We direct attention to the following tabular statement:—

"RETURN of the Number of ADMISSIONS, DISCHARGES, and DEATHS in *Dublin Lunatic Asylums* during the Years ending 31st March, 1850, and 31st March, 1851.

| — | Year ending 31st March, 1850. | | | Year ending 31st March, 1851. | | |
|---|---|---|---|---|---|---|
| | Males. | Females | Total. | Males. | Females | Total. |
| Admitted . . . . . . . . . . | 451 | 438 | 889 | 443 | 457 | 900 |
| Discharged during the year—Cured . | 227 | 211 | 438 | 206 | 228 | 434 |
| " " Improved | 36 | 56 | 92 | 41 | 61 | 102 |
| " " Not cured | 17 | 10 | 27 | 35 | 21 | 56 |
| " " Incurable | 22 | 21 | 43 | 18 | 29 | 47 |
| Total Discharged . . . . . | 302 | 298 | 600 | 300 | 339 | 639 |
| Died during the year . . . . . . . | 173 | 150 | 323 | 159 | 106 | 265 |
| Number of Inmates on 31st March . | 1382 | 1329 | 2711 | 1386 | 1362 | 2748 |

This Return includes the patients in Island-bridge, belonging to the Metropolitan District.

"The preceding table shows an increase of 73 cures over the two corresponding years of our last report, and a diminution of 140 deaths, the admissions being respectively within the same periods 1861 and 1789."

Only ONE case of suicide is recorded in the Report—and, strange to observe, it is the only instance that occurred in the public asylums of Ireland for many years! In referring to the mortality from cholera, the inspectors observe :—

"Although cholera was very prevalent and fatal throughout the kingdom during the summer and autumn of '49, with the exception of twenty-four deaths at the Limerick asylum, in the course of a week, and two cases elsewhere, we have to record no mortality from that disease. Its appearance was equally sudden and inexplicable at Limerick, attacking six-and-twenty patients within a few hours—the corridors in which it broke out, and to which it was principally confined, being as well-ventilated and orderly as any in the establishment, and the victims themselves of various ages, previously in good health. We received reports at the time from the Carlow and other asylums, that some of the inmates had been affected with the usual premonitory symptoms; but by an immediate alteration of dietary, so as to increase their physical comforts, no ill consequences resulted."

It appears that the fund resulting from the employment of the patients amounted to 2860*l.* In referring to the influence of religion on the insane, the report, we are pleased to say, confirms our own opinion of its salutary influence on those afflicted with derangement of mind. It is observed—

"That the regular attendance of chaplains produces a salutary influence on the insane. No doubt the introduction of devotional subjects, where a pre-disposition to excitement on such topics is observable, would be ill-timed and erroneous, but in all other cases we are of opinion religious conversation and observances tend both to control and soothe."

The inspectors advocate the adoption of the continental system, and observe, that, had the proposition of the Chancellor of the Exchequer with reference to the support of lunatic poor from the consolidated fund been adopted, much difficulty would be obviated on this head, and a uniform system could with the utmost facility be established in detail, for the maintenance and management of hospitals for the insane, as, under existing circumstances, the governors, however liberally disposed in the discharge of their trust as guardians of the public purse, are naturally cautious of innovations, no matter how advisable, when a direct and increased expenditure is involved. We believe were the government of this country to adopt a system prevalent abroad, and place institutions for the cure and treatment of lunatics (whose safeguard is of such importance to the general well-being of society), if not entirely, in part, at least, on state support, the result would be found satisfactory and economical in the end; whilst the sum thus saved to the community from the previous maintenance of asylums would become available for general medical charities. As a case in point, we shall instance the city of Dublin, which averages to the Richmond district asylum 3400*l.* a year, a sum which, if withdrawn from that establishment, would admit its equivalent of taxation for the benefit of the common hospitals of the metropolis.

We now approach the consideration of that part of the official document relating to the condition of criminal lunatics, from which we purpose borrowing largely.

The establishment of a central asylum for the reception and custody of lunatics charged with offences in Ireland, was provided by a special Act, 8 and 9 Vic., cap. 107, and at the same time a parliamentary grant of 6000*l.* was voted for the objects; immediately after which a site, possessing a cheerful, open, and healthy aspect, was selected near Dundrum, in the county of Dublin, about three miles from the city at its southern side. The purchase of fourteen statute acres was effected by the Commissioners of Public Works, at a cost of 2300*l.*, and the buildings, commenced in 1846, were finally terminated in July, 1850, when the establishment, with an accommodation for eighty male and forty female lunatics, was placed under the control of the inspectors, who were directed to draw up a code of rules and regulations for its maintenance and management. Prior to the admission of any patients, a list of all the criminal lunatics then in confinement was made out, whether in gaols or district asylums, the total aggregate of whom amounted to 178 : from this number the inspectors selected, with a detailed history of their offences, eighty-four individuals, who, *in limine,* seemed peculiarly proper objects to come within the spirit of the Act. It would have been impossible, from the disparity of the cases that could be accommodated, with the total above named, to embrace all the criminal lunatics; the inspectors confined themselves to lunatics charged with offences which, if committed by sane persons, would involve a punishment either by death, transportation, or an imprisonment for two years. Culprits guilty of minor offences, such as petty larcenies, attempted assaults, the use of threatening language, and who, generally speaking, for want of bail, were originally committed to prison, were not looked upon as suitable cases for transmission to the central asylum; at the same time exceptions were made of certain parties, though barely coming within the term criminal, in consequence of their dangerous and uncontrollable propensities; such individuals being, with the sanction of the lord-lieutenant, and by warrant, duly transferred to Dundrum. As might have been expected on a revision of the criminal lunatics, there were some charged with serious offences, who, under treatment, had perfectly recovered. These cases were submitted to the lord-lieutenant's benevolent consideration, and at the inspectors' recommendation, he was pleased, under certain restrictions, to restore the parties to liberty: the first was one of infanticide, perpetrated about fifteen years before by a married woman, whilst labouring under puerperal mania, on her own child. She had been similarly affected on two previous occasions, but fortunately without any bad results; for the last twelve years of her residence in the Belfast Asylum, she was perfectly sane, conducting herself in an exemplary manner. As the cause of her malady was clearly physical, and her then period of life protected her against its recurrence, his excellency afforded this female an immediate pardon, and directed she should be placed under the protection of her family. The next instance was that of a man, who killed his companion in a paroxysm of maniacal excitement in the year 1831: he was tried and acquitted on the plea of insanity, and soon after removed to the Carlow Asylum. Subsequent to his recovery, which took place in the course of a year, he employed himself as steward on the grounds, and in aiding the attendants in their various charges; on the erection of the Dundrum Asylum, he memorialized to be allowed to remain in Carlow, 'where all his associations were centered,' or to emigrate. As the former request could not be legally acceded to, his excellency being satisfied as to the character of the man, and the securities entered into that he would not return to this country, gave him permission to leave. He sailed from Liverpool for New York, about a fortnight after was shipwrecked on the north-west coast of Ireland, lost whatever property he possessed, and, narrowly escaping with his life, came up to Dublin and placed himself under the control of the inspectors, and is now awaiting further arrangements. There were two other cases of homicide, the lunatics being for a series of years perfectly restored to reason, useful, and well-behaved, whom his excellency has liberated; the individuals, one binding himself under heavy recognizance, to dwell no longer in the locality where the offence was committed; and the other to leave the kingdom altogether, and emigrate with his family.

In the prudent benevolence of the lord-lieutenant's decision, the inspectors most respectfully and cordially concurred. They do so the more from the strong feelings they entertain with regard to the plea of lunacy on capital indictments; for, on the one hand, as nothing appears to them so utterly injurious to the cause of justice and humanity as an excuse for crime under the garb of insanity, when insanity is not distinctly proved to exist; so, on the other, they think that mercy may make some allowance for the act of the unhappy maniac, however lamentable it may have been, and dissever it when he is permanently restored to reason from its expiation by a perpetual confinement, provided such can be done with safety to the community at large, and without offending the reasonable prejudices of the public.

The Report observes, as the result of a minute examination into the many real or simulated cases of criminal insanity that have come under the notice of the authorities, that ultimately no greater damage can be engendered to the very object it would desire to serve, than an over-stretched morbid disposition to render lunacy the protector, as it were, of crime, and thereby to acquit prisoners in the dock without a rigid inquiry, and the clearest evidence of the correctness of the plea; if there are extenuating circumstances connected with the psychological condition of the accused, they are legitimate subjects to be considered in meting out the after-punishment, but certainly not in the first instance for an unqualified acquittal.

Several cases of feigned insanity to defeat the ends of justice came under the official cognizance of the inspectors. The first is that in which a young woman murdered her husband, and who from the time of her committal to that of her arraignment, a period of about three months, simulated insanity, with occasional lucid intervals, but of very short duration—she was tried, convicted, and executed. The determination and fixity of purpose displayed by her was extraordinary; she seemed proof against experiment; and though secretly watched for weeks, never, even in private, deviated from a line of deception; she frequently lay on the floor from night to morning, without the slightest change of attitude. Her demeanour and language all through were the most incoherent; still there was about her an incongruity of symptoms which impressed Dr. Jacob, physician to the gaol, as well as the inspectors at their several visitations, that she was feigning. Subsequent to condemnation she became quite resigned to her fate, and acknowledged the crime of which she had been accused, as well as her attempts at deception. The chief difficulty which presented itself in the investigation of the case, arose from the circumstance that a near relative of the unfortunate woman died in a lunatic asylum. With the history of the next case his excellency took a deep interest. We believe that the moral influence of the law was fully vindicated in this instance; the individual not escaping capital punishment on the simple plea of insanity, but subsequent to sentence from circumstances which his excellency considered a sufficient warrant to commute the extreme penalty to transportation for life. We refer to the case of William Quinlan, whose abandonment of self-control and recklessness of conduct led to his discharge from the regiment he served in abroad, and to his transference, for a few months, to the Military Asylum at Fortpitt, on leaving which he returned to his native county, Tipperary, where, for years, his sanity was never questioned. Notoriously a profligate and vicious character, he, with two accomplices, was bribed to the preconcerted murder of four bailiffs—a plea of lunacy was attempted. In the dock he assumed a mixed air of levity and folly, and after condemnation, of such maniacal excitement, that the clergy of his persuasion refused to attend him. A memorial was consequently forwarded on his behalf, and officers under whom he had served expressed personally, and by letter, to his excellency, their belief that the convict was not of sound mind. On mature consideration of the antecedents of this man's history, his sentence was commuted to transportation. During his sojourn in the gaol of Clonmel, and up to the period when he was informed his life would be spared, his demeanour was that of a violent lunatic; subsequently he became amenable and tranquil.

If, on the one hand, the severest penalty of the law could not consistently be inflicted on this convict, on the other, it is equally evident he was not a fit subject for a lunatic asylum, for had he not committed the serious crime which brought him to trial, he might to the present moment be leading his usual life of reckless dissipation, as no authority could interfere with his civil liberty on the score of insanity, and in our opinion the moral result would be alike unfavourable were he in the first instance acquitted on the plea of lunacy, or, subsequent to conviction, made the inmate of an asylum; in any locality he must prove a difficult subject to control. The third and last case is that of John Grady, under sentence of transportation, and confined for the last year at Spike Island depôt; this man, formerly a respectable landholder in the county of Limerick, murdered his wife and servant in February, 1847. Subsequent to his committal to prison, which took place shortly afterwards, he appeared quite insane, and attempted suicide. When called on to plead at the ensuing spring assizes, as two medical gentlemen expressed their belief that he was of unsound mind, the order usual on such occasions was made by the bench, and in due course he was transferred to the district asylum, where the inspectors frequently visited him, always, however, under the impression that his madness was simulated. By degrees he came to himself, and on testing his recovery, if such it might be termed, by a residence of over sixteen months in the institution, the inspectors submitted the case to Sir Thomas Redington, by whose directions he was recommitted, in December, 1849, to gaol, and arraigned at the next assizes. After an imposing trial, which lasted over fourteen hours, and at which, though the medical testimony varied materially in regard to the mental condition of the prisoner, not a doubt existed as to his perpetration of the crime under the most appalling circumstances; he was found guilty, and left for execution; a sentence which was commuted, the ends of justice having been fully attained by the conviction of this unhappy man.

The criminal lunatics transferred to the Central Asylum at its opening amounted to eighty-four, of whom forty-three were homicides. At the last assizes three additional cases charged with murder were admitted; one of the parties is demented, the other two, though at present sane, are not the less legitimate subjects for the institution; the offences of which they were accused being committed during the maniacal excitement which so often supervenes on epilepsy, and to which it appeared on evidence they were subject, though at distant intervals. As no species of insanity is more dangerous than that combined with the above disease, it at all times requires the most cautious supervision; and when a disposition to violence exists, even in the absence of a serious criminal offence, on no account would the neglect of a proper control be countenanced, by allowing such persons at large. The difference between the number of homicides, and the aggregate, ninety-one, at present confined in the institution, is composed of individuals charged with arson, aggravated assaults, and, as before observed, with minor offences, but who evince mischievous or malignant tendencies.

Amongst the many interesting cases in the asylum, there is one alone to which we shall refer; unparalleled, as it is, in the annals of criminal lunacy, not alone from the extent and frightful character of the act itself, but, perhaps, still more from the infatuated credulity of the victims. It is that of Captain S., who, on his return from the West Indies, murdered seven of his crew at sea. It appeared in evidence, that on leaving Barbadoes he laboured under great mental depression, and a day or two afterwards accused his mate of exciting the sailors to mutiny.

The report of the case is as follows:—

"During the voyage he scarcely ever took rest, and for the last two nights lay on a sofa, with a brace of loaded pistols and cutlass by his side, apprehensive of being attacked. When in sight of the Cork coast, he threatened, on arriving in port, to prosecute the whole crew. The mate remonstrated, when Captain S. replied, 'Show your obedience by allowing me to bind you down on deck.' The man consented; he then called on the second mate, pointed out what had been done, and desired him to follow the example—he did so; in fact, seven of eight individuals permitted themselves to be fastened to the deck with ropes; when thus incapable of defence, he deliberately murdered them in various ways; the eighth escaped into the hold, and was wounded there by a pistol-shot. At the moment, a pilot-boat ran alongside; Captain S. jumped overboard, was saved, and brought into Cork harbour with his vessel. He is now a religious monomaniac, generally very tranquil and rational, subject however, at intervals, to maniacal paroxysms, the forerunner to which is an access of piety, with a recurrence to the phraseology of his former profession. He is still impressed with the belief that the crew meditated mutiny and his death."

On the subject of private asylums, it is said—

"Independent of the duty imposed on us by the legislature, we feel more than an ordinary degree of interest in the well-being of these establishments, the more particularly as, previous to the passing of the above Act, there was no proper system of inquiry or that inspection which is so advisable to be maintained in all asylums appropriated to the reception and treatment of lunatics belonging to the better classes of society. We have consequently directed a particular attention to this branch of our department, with the hope of rendering it as perfect as possible, and we are happy at being enabled to state that our efforts for the improvement of private licensed houses, have, up to the present time, been attended with considerable success.

"In our last Report we made mention of structural alterations intended to afford lightsome and airy apartments, as well as of various domestic arrangements in keeping with the previous station in life, and tending to the social enjoyments of their several occupants. As these and other improvements have been in a great measure effected, we anticipate, with the assistance of the proprietors, who as a body evince a strong disposition to do all that is required of them, that, at no distant period, the private asylums of this country will be placed on a most satisfactory footing.

"On our several visitations to each of these institutions, we minutely addressed ourselves to the various inquiries directed by the Act, and, on the whole, are justified in stating that its provisions have been strictly observed. Occasionally complaints are preferred by patients to us personally or in writing; the great majority of them, however, on investigation would appear to be altogether fanciful in their origin, arising principally from delusions connected with family feuds, conspiracies, and a supposed ill-usage consequent thereon. In some rare instances, we felt called on to interfere, and however disagreeable remonstrance with the parties might have been, we did not hesitate to require such a course as the peculiar circumstances of the cases may have pointed out.

"We have further examined into certain cases to which our attention was directed, and on which special reports were forwarded to your Excellency or the Lord Chancellor.

"With reference to the non-coercion system, our experience is strongly in its favour; for testing this mode of treatment as maintained in our private asylums, where, consistent with safety, an ample latitude of freedom is allowed, and restraint, if not altogether abolished, is reduced to the very lowest degree, and then so mild as not to irritate the patient, we cannot but arrive at the satisfactory conclusion that the more lunatics are treated and respected as sane persons, the more amenable they become, and the greater their chance of ultimate recovery. There is a type of mental disease, by no means uncommon in private asylums, and to which we would here refer, the persons affected by it enjoying healthy intervals of longer or shorter duration. Aware that however sane in act and conversation such patients may be within the precincts of the institution, the excitement of every-day life would tend to bring on a recurrence of the disorder, we do not consider it advisable to interfere, save in cases where a decided improvement, with long intermissions, has already manifested itself. Although our personal experience has not, up to the present, furnished us with any case involving difficulties, either as regards the social rights of individuals labouring under the particular affection to which we have just alluded, the disposition of their property—or an immunity from the responsibility attaching to crime—very grave considerations in a medico-legal point of view may nevertheless arise therefrom. To obviate as much as possible such contingencies, we feel satisfied that too much exactitude cannot be observed by the attendants at all times, and by the inspectors on their several visitations, in examining into and noting the condition and general conduct of such individuals.

"The mental and bodily health of the inmates of private licensed houses during the past two years, afford subject of much gratification, the former as exemplified by the per centage of cures, and the latter by a mortality considerably less than has occurred within a similar period since the passing of the Act. Occupations and means of amusement, if not quite to the extent we could desire, are still fairly provided for in them, whilst a distinct classification in some, and a partial only in others, is kept up, but, as we have formerly remarked to your excellency, the greatest inconvenience is felt in this respect, where such establishments have not been originally constructed for the reception of lunatics.

"We have unceasingly urged upon the various proprietors the importance of making a judicious selection of attendants, persons of good character and education; feeling satisfied that they contribute in no ordinary degree to the recovery, as well as the happiness, of those committed to their charge, and over whom they should exercise a well regulated moral influence. We are gratified to observe that the system of registry introduced by us in 1848, has operated most beneficially in these establishments.

"With regard to the dietary, we have had no reason to complain either of the quality or quantity of the food supplied, but we regret that in two or three licensed houses there is a want of neatness and comfort in the manner in which the meals are served. We trust, however, that through constant attention to this subject, and the inculcation of habits of order and cleanliness, a speedy reformation will take place. In our inquiries as to the supply of wearing apparel by the friends of patients, answers have been for the most part satisfactory; in this particular we have observed a decided improvement during the past year.

"Facilities of attendance at religious worship are afforded to the patients, who in some instances regularly visit on Sundays and other days of devotion their respective churches. Generally speaking, however, clergymen attend to officiate in the asylums.

"The fourteen houses licensed for the reception of lunatics in Ireland, and containing an aggregate of four hundred and forty-six inmates, are situated in the counties of Dublin, Armagh, the Queen's, Waterford, and Cork, and with three exceptions belong to medical proprietors.

"The following table presents a summary of the principal statistics connected with these establishments since the date of our last public Report:—

| | Males. | Females. | Total. |
|---|---|---|---|
| In asylums 31st December, 1848 | 245 | 187 | 432 |
| Admitted in 1849 and 1850 | 159 | 104 | 263 |
| Discharged in 1849 and 1850:— | | | |
| Cured | 56 | 36 | 92 |
| Improved | 57 | 36 | 92 |
| Not cured | 14 | 15 | 29 |
| Died | 26 | 15 | 41 |
| In asylum, 1851 | 251 | 195 | 446 |

Social Condition of Patients admitted in 1849-50:—

| | | |
|---|---|---|
| Married | | 146 |
| Unmarried | | 217 |
| Total admissions | | 263 |
| Army and Navy | 20 | |
| Clerical | 13 | |
| Legal | 7 | |
| Medical | 7 | |
| Merchants, or in Trade | 57 | |
| Clerks and Teachers | 21 | |
| Landholders | 20 | |
| No occupation | 118 | |

We copy the following tabular statements from the Report before us, as they embody points of great interest to all connected with the management of asylums and the treatment of the insane:—

### DISTRICT LUNATIC ASYLUMS.

### APPENDIX No. I.—Showing the Number of ADMISSIONS and DISCHARGES during the Year ended 31st March, 1850.

| Asylums. | In Asylums on 31st March, 1849. | | | Admitted from 31st March, 1849, to 31st March, 1850. | | | Discharged during the Year. | | | | | | | | | | | | | | | Died during the Year. | | | In Asylum on 31st March, 1850. | | |
|---|---|---|---|---|---|---|---|---|---|---|---|---|---|---|---|---|---|---|---|---|---|---|---|---|---|---|---|
| | | | | | | | Cured. | | | Improved. | | | Not Cured. | | | Incurable. | | | Total Discharged. | | | | | | | | |
| | M. | F. | Total. | M. | F. | Total. | M. | F. | Total. | M. | F. | Total. | M. | F. | Total. | M. | F. | Total. | M. | F. | Total. | M. | F. | Total. | M. | F. | Total. |
| Armagh . . | 67 | 65 | 132 | 21 | 24 | 45 | 4 | 9 | 13 | 5 | 5 | 10 | 2 | 2 | 4 | — | — | — | 11 | 16 | 27 | 4 | 11 | 15 | 73 | 62 | 135 |
| Belfast . . | 150 | 114 | 264 | 57 | 63 | 120 | 24 | 28 | 52 | 11 | 10 | 21 | — | — | — | — | — | — | 35 | 38 | 73 | 25 | 18 | 43 | 147 | 121 | 268 |
| Londonderry | 117 | 106 | 223 | 27 | 49 | 76 | 13 | 17 | 30 | 1 | 4 | 5 | — | — | — | — | — | — | 14 | 21 | 35 | 16 | 29 | 45 | 114 | 105 | 219 |
| Richmond . | 137 | 150 | 287 | 69 | 49 | 118 | 33 | 17 | 50 | 2 | 5 | 7 | 2 | 1 | 3 | 21 | 20 | 41 | 58 | 43 | 101 | 10 | 11 | 21 | 138 | 145 | 283 |
| Carlow . . | 108 | 90 | 198 | 27 | 29 | 56 | 11 | 13 | 24 | 4 | 6 | 10 | — | 2 | 2 | — | — | — | 15 | 21 | 36 | 10 | 7 | 17 | 110 | 91 | 201 |
| Ballinasloe . | 159 | 141 | 300 | 44 | 37 | 81 | 13 | 15 | 28 | 1 | — | 1 | 1 | 2 | 3 | — | 1 | 1 | 15 | 18 | 33 | 28 | 8 | 36 | 160 | 152 | 312 |
| Limerick . . | 151 | 160 | 311 | 80 | 41 | 121 | 54 | 24 | 78 | 5 | 3 | 8 | 10 | — | 10 | — | — | — | 69 | 27 | 96 | 7 | 5 | 12 | 155 | 169 | 324 |
| Maryborough | 98 | 99 | 197 | 28 | 26 | 54 | 20 | 17 | 37 | — | — | — | — | — | — | — | — | — | 20 | 17 | 37 | 9 | 10 | 19 | 97 | 98 | 195 |
| Clonmel . . | 64 | 69 | 133 | 19 | 11 | 30 | 11 | 13 | 24 | — | — | — | 1 | 1 | 2 | — | — | — | 12 | 14 | 26 | 7 | 2 | 9 | 64 | 64 | 128 |
| Waterford . | 51 | 66 | 117 | 21 | 41 | 62 | 14 | 24 | 38 | 1 | 13 | 14 | — | — | — | 1 | — | 1 | 16 | 37 | 53 | 9 | 3 | 12 | 47 | 67 | 114 |
| Cork . . . | 212 | 198 | 410 | 58 | 68 | 126 | 30 | 34 | 64 | 6 | 10 | 16 | 1 | 2 | 3 | — | — | — | 37 | 46 | 83 | 36 | 35 | 71 | 197 | 185 | 382 |
| Total . | 1314 | 1258 | 2572 | 451 | 438 | 889 | 227 | 211 | 438 | 36 | 56 | 92 | 17 | 10 | 27 | 22 | 21 | 43 | 302 | 298 | 600 | 161 | 139 | 300 | 1302 | 1259 | 2561 |

### APPENDIX No. II.—Showing the Number of ADMISSIONS and DISCHARGES during the Year ended 31st March, 1851.

| Asylums. | In Asylums on 31st March, 1850. | | | Admitted from 31st March, 1850, to 31st March, 1851. | | | Discharged during the Year. | | | | | | | | | | | | | | | Died during the Year. | | | In Asylums on 31st March, 1851. | | |
|---|---|---|---|---|---|---|---|---|---|---|---|---|---|---|---|---|---|---|---|---|---|---|---|---|---|---|---|
| | | | | | | | Cured. | | | Improved. | | | Not Cured. | | | Incurable. | | | Total Discharged. | | | | | | | | |
| | M. | F. | Total. | M. | F. | Total. | M. | F. | Total. | M. | F. | Total. | M. | F. | Total. | M. | F. | Total. | M. | F. | Total. | M. | F. | Total. | M. | F. | Total. |
| Armagh . . | 73 | 62 | 135 | 26 | 28 | 54 | 6 | 14 | 20 | 6 | 3 | 9 | 10 | 4 | 14 | — | — | — | 22 | 21 | 43 | 10 | 5 | 15 | 67 | 64 | 131 |
| Belfast . . | 147 | 121 | 268 | 69 | 71 | 140 | 33 | 48 | 81 | 11 | 18 | 29 | — | — | — | — | — | — | 44 | 66 | 110 | 22 | 7 | 29 | 150 | 119 | 269 |
| Londonderry | 114 | 105 | 219 | 53 | 50 | 103 | 31 | 31 | 62 | 5 | 2 | 7 | 4 | 3 | 7 | — | 1 | 1 | 40 | 37 | 77 | 11 | 11 | 22 | 116 | 107 | 223 |
| Richmond . | 138 | 145 | 283 | 53 | 60 | 113 | 18 | 15 | 33 | 2 | 8 | 10 | 9 | 6 | 15 | 15 | 25 | 40 | 44 | 54 | 98 | 14 | 5 | 19 | 133 | 146 | 279 |
| Carlow . . | 110 | 91 | 201 | 33 | 37 | 70 | 17 | 19 | 36 | 6 | 6 | 12 | 2 | 1 | 3 | — | — | — | 25 | 26 | 51 | 11 | 12 | 23 | 107 | 90 | 197 |
| Ballinasloe . | 160 | 152 | 312 | 44 | 32 | 76 | 18 | 13 | 31 | 2 | 1 | 3 | 1 | — | 1 | 3 | 1 | 4 | 24 | 15 | 39 | 18 | 19 | 37 | 162 | 150 | 312 |
| Limerick . . | 155 | 169 | 324 | 59 | 36 | 95 | 29 | 22 | 51 | 3 | 2 | 5 | 3 | 1 | 4 | — | 2 | 2 | 35 | 27 | 62 | 11 | 6 | 17 | 168 | 172 | 340 |
| Maryborough | 97 | 98 | 195 | 24 | 24 | 48 | 9 | 15 | 24 | 3 | 3 | 6 | 4 | 1 | 5 | — | — | — | 16 | 19 | 35 | 10 | 6 | 16 | 95 | 97 | 192 |
| Clonmel . . | 64 | 64 | 128 | 18 | 21 | 39 | 13 | 8 | 21 | 1 | — | 1 | 2 | 4 | 6 | — | — | — | 16 | 12 | 28 | 4 | 3 | 7 | 62 | 70 | 132 |
| Waterford . | 47 | 67 | 114 | 17 | 25 | 42 | 9 | 8 | 17 | 1 | 13 | 14 | — | — | — | — | — | — | 10 | 21 | 31 | 7 | 3 | 10 | 47 | 8 | 115 |
| Cork . . . | 197 | 185 | 382 | 47 | 73 | 120 | 23 | 35 | 58 | 1 | 5 | 6 | — | 1 | 1 | — | — | — | 24 | 41 | 65 | 26 | 17 | 43 | 194 | 200 | 394 |
| Total . | 1302 | 1259 | 2561 | 443 | 457 | 900 | 206 | 228 | 434 | 41 | 61 | 102 | 35 | 21 | 56 | 18 | 29 | 47 | 300 | 339 | 639 | 144 | 94 | 238 | 1301 | 1283 | 2584 |

The Cases transferred from the Richmond Asylum to Island Bridge as incurable, are not enumerated in the above Return of Discharges.

### Appendix No. III.—Return showing the Names and Salaries of the Principal Officers of the *Several District Lunatic Asylums*, March, 1851.

| Asylums. | Visiting Physician. | Resident Physician and Superintendent* Manager. | Matron. | Protestant Chaplain. | Roman Catholic. | Apothecary. | Clerks and Store-keepers. |
|---|---|---|---|---|---|---|---|
| | Name and Salary. | Name and Salary. | Name and Salary. | Name and Salary. | Name and Salary. | Name and Salary. | Name and Salary. |
| | £ | £ | £ s. d. | £ | £ | £ | £ |
| Armagh . . | T. Cumming, M.D. . 100 | Thomas Jackson . 200 | M. Jackson . 50 0 0 | None. | None. | Vacant. | W. Renning . . 20 |
| Belfast . . | H. M'Cormack, M.D. 100 | R. Stewart, M.D.* 200 | M. F. Stewart 50 0 0 | None. | None. | J. S. Mulholland . 25 | Robert Lamont . 40 |
| Londonderry | Francis Rogan, M.D. 100 | David Cluff . . . 200 | Eliza Cluff . 50 0 0 | Rev. M. Wilson . 25<br>Rev. Dr. Denham, Pres. . . . . 25 | Rev. H. Nugent 25 | Charles Morton . 30 | Robt. Hamerton . 30 |
| Richmond | J. Mollan, M.D. 168*l.* 9*s.* 4*d.*<br>R. Tuohill, M.D., physician extraordinary, acting without salary<br>J. Hughes, surgeon . 50 | Samuel Wrigley 250 | C. Wrigley . 55 7 8 | Rev. G. Black . . 50 | Rev. J. Falkner 50 | P. Beatty, 27*l.* 13*s.* 8*d.* | J. Harley . . . 50 |
| Carlow . . | Thos. O'Meara, M.D. 100 | M. E. White, M.D.* 200 | L. Parsons . 100 0 0 | Rev. F. F. T. Trench 25 | Rev. D. M'Carthy 25 | H. Montgomery . 25 | Timothy Brenan . 40 |
| Ballinasloe . | Wm. Colahan, M.D. 125 | J. B. M'Kiernan . 200 | M. A. Callan 60 0 0 | Rev. H. Walker . 40 | Rev. L. Dillon 40 | J. Callan . . . 30 | Thomas Callen . 55 |
| Limerick . . | D. O'Callaghan, M.D. 150 | R. Fitzgerald, M.D.* 210 | A. M. Sleeman 70 0 0 | Rev. B. Jacob . . 50 | Rev. J. Bunton 50 | T. & J. Bouchier . 30 | Patrick M'Donnell 60 |
| Maryborough | John Jacob, M.D. . . 100 | T. C. Burton, M.D.* 250 | Eliza Abbott 85 0 0 | Rev. T. Harpur . 25 | Rev. M. O'Connor 25 | Thos. Pilsworth . 30 | James Vanston . 40 |
| Clonmel . . | Wm. J. Sheill, M.D. 100 | Jas. Flynn, M.D.* 200 | Ellen Crofton 70 0 0 | Vacant. | — | Richard Graham 30 | Edward O'Neill . 55 |
| Waterford . | Wm. Connolly, M.D. 100 | John Dobbs . . . 200 | C. Ronayne . 70 0 0 | Rev. Robert Bell . 25 | Rev. P. Wall. . 25 | John Mackesy . . 30 | Thomas Keary . 45 |
| Cork . . | T. Power, physician 175<br>S. Hobart, surgeon . 100 | Eugene O'Neill . 200 | M. Merrick 60 0 0<br>Martha Smith, asst. m. . 50 0 0 | Rev. C. H. Clifford 50 | Rev. M. O'Sullivan . . . . 50 | W. T. Jones . . 25 | W. Connell, clerk 25<br>R. Thorp, store-keeper . . . 35 |

### Return of the Names and Salaries of the Officers of the *Central Lunatic Asylum, Dundrum.*

| Visiting Physician. | Salary. | Resident Physician and Governor. | Salary. | Matron. | Salary. | Protestant Chaplain. | Salary. | Roman Catholic. | Salary. | Clerk and Store-keeper. | Salary. |
|---|---|---|---|---|---|---|---|---|---|---|---|
| | £ s d. | | £ s. d. | | £ s. d. | | £ s. d. | | £ s. d. | | £ s. d. |
| Robert Harrison, M.D. | 150 0 0 | W. Corbet, M.D. . . | 240 0 0 | Sarah M'Donnell . . | 80 0 0 | Rev. Charles Stanford | 30 0 0 | Rev. Dr. Ennis . | 60 0 0 | John Royse . . | 100 0 0 |

We congratulate the Irish government, the Irish nation, and the friends of humanity, on the progress which, under a wise system of executive government, has been made in Ireland of late years in the establishment of a liberal and enlightened mode of treating the insane. The report of the official inspectors is an able state document. It is evidently drawn up with great care, and merits the patient attention and study of all interested in the advancement of psychological science.

---

